# Talin1 targeting potentiates anti-angiogenic therapy by attenuating invasion and stem-like features of glioblastoma multiforme

**DOI:** 10.18632/oncotarget.4835

**Published:** 2015-08-20

**Authors:** Wonyoung Kang, Sung Heon Kim, Hee Jin Cho, Juyoun Jin, Jeongwu Lee, Kyeung Min Joo, Do-Hyun Nam

**Affiliations:** ^1^ Samsung Biomedical Research Institute, Samsung Medical Center, Seoul, Korea; ^2^ Department of Health Sciences and Technology, SAIHST, Sungkyunkwan University, Seoul, Korea; ^3^ Department of Neurosurgery, Samsung Medical Center, Sungkyunkwan University School of Medicine, Seoul, Korea; ^4^ Department of Anatomy and Cell Biology, Sungkyunkwan University School of Medicine, Seoul, Korea; ^5^ Department of Stem Cell Biology and Regenerative Medicine, Lerner Research Institute, Cleveland Clinic, Cleveland, OH, USA

**Keywords:** glioblastoma multiforme, anti-angiogenic therapy, bevacizumab-resistance, Talin1, patient derived xenograft models

## Abstract

Glioblastoma multiforme (GBM) possesses florid angiogenesis. However, the anti-angiogenic agent, *Bevacizumab*, did not improve overall survival of GBM patients. For more durable anti-angiogenic treatment, we interrogated resistant mechanisms of GBM against *Bevacizumab*. Serial orthotopic transplantation of *in vivo Bevacizumab*-treated GBM cells provoked complete refractoriness to the anti-angiogenic treatment. These tumors were also highly enriched with malignant phenotypes such as invasiveness, epithelial to mesenchymal transition, and stem-like features. Through transcriptome analysis, we identified that Talin1 (TLN1) significantly increased in the refractory GBMs. Inhibition of TLN1 not only attenuated malignant characteristics of GBM cells but also reversed the resistance to the *Bevacizumab* treatment. These data implicate TLN1 as a novel therapeutic target for GBM to overcome resistance to anti-angiogenic therapies.

## INTRODUCTION

Glioblastoma multiforme (GBM) is the most aggressive and infiltrative primary brain tumor. GBM is a well-vascularized tumor with florid angiogenesis. Median survival days of the patients diagnosed with GBM remains less than 15 months despite maximal therapeutic treatments [1–3]. High degree of endothelial proliferation is one of criteria for GBM diagnosis. In addition, GBM cells express large amounts of vascular endothelial growth factor (VEGF) and other angiogenic molecules that promote formation of new and permeable vessels [4–8]. These suggest that inhibition of angiogenesis might be an effective therapeutic strategy against GBM, and indeed various anti-angiogenic therapeutic strategies have been tested in GBM.

Bevacizumab is a humanized monoclonal antibody that selectively binds with VEGF to inhibit interaction between VEGF and its receptors VEGFRs [9, 10]. A few phase 2 clinical trials of in patients with recurrent GBM showed transient but dramatic responses, leading to Food and Drug Administration (FDA) approval for the treatment of recurrent GBMs. Recent large-scale clinical trials in patients with newly diagnosed GBM failed to deliver prolongation of overall survival and confirmed transient therapeutic effects of Bevacizumab [11–17].

Resistance to anti-angiogenic therapy appears to be emerged by adaptive mechanisms. GBM tumors after anti-angiogenic therapy often revealed enhancement of more invasive and infiltrative phenotypes and inevitably relapsed [14, 16, 18]. Understanding the molecular mechanisms of the anti-angiogenic therapy resistance is a critical unmet need to improve the patient outcome. Here, we describe our systematic interrogation of *Bevacizumab*-resistance and identification of the cytoskeleton protein Talin1 (TLN1) as a key regulator of *Bevacizumab*-resistance. Furthermore, by utilizing patient-derived GBM xenografts and serial transplantation models, we interrogated functional roles of TLN1 in stem cell features, invasion, and *Bevacizumab*-resistance of GBM.

## RESULTS

### Establishment of the *Bevacizumab*-resistant GBM models

**Figure 1:**
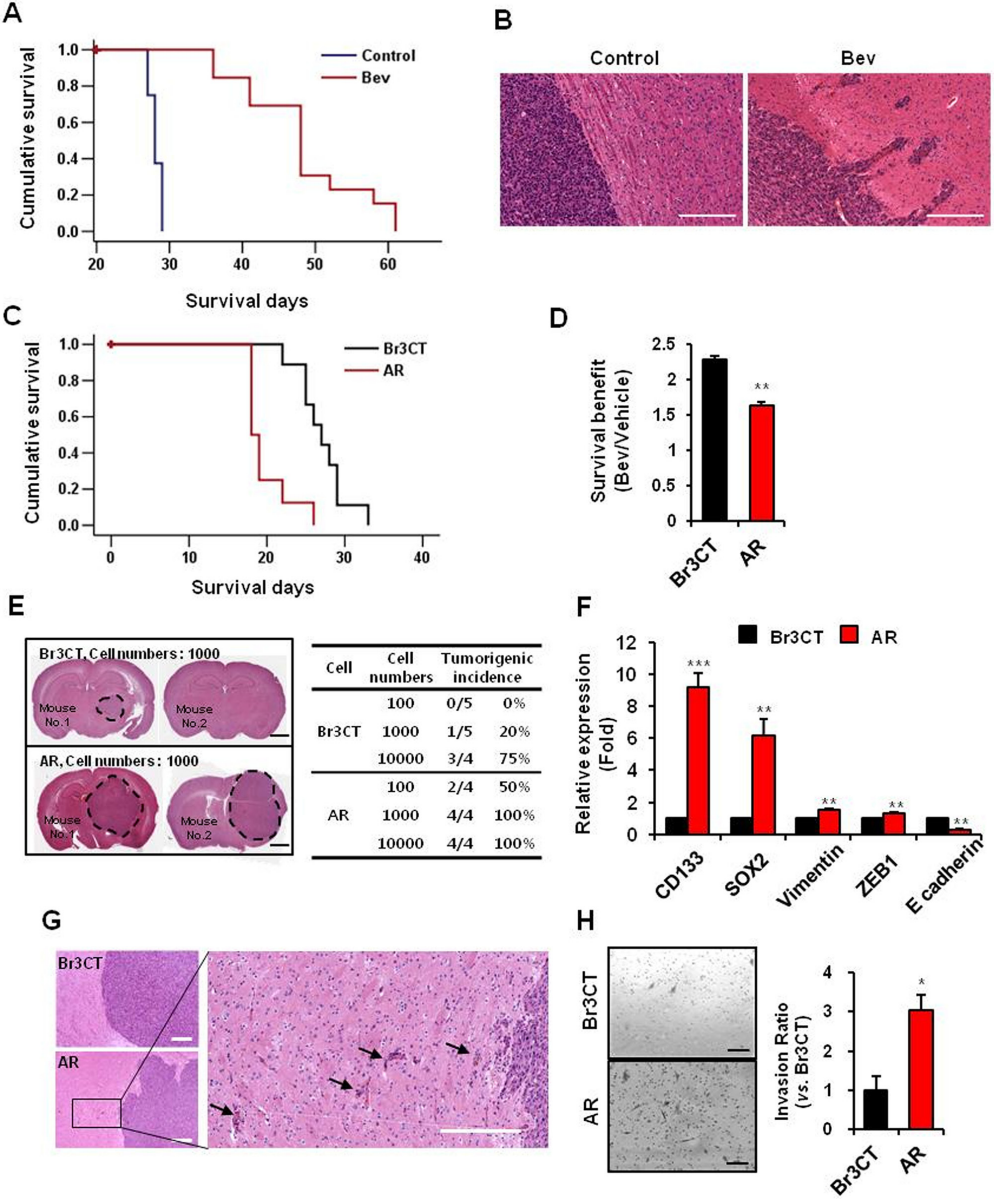
Bevacizumab treatment increase malignant progress of GBM **A.** Kaplan-Meier survival curves of mice orthotopically implanted with U87MG cells treated with or without bevacizumab. *p* < 0.001. **B.** H&E staining of brains of mice treated with or without bevacizumab. Bar represents 200 micron. **C.** Kaplan-Meier survival curves of mice orthotopically implanted with Br3CT or AR. *p* < 0.001. **D.** Survival benefit calculated by increasing survival rate by bevacizumab treatment in mice orthotopically implanted each cells. Data are means ± SE. **p < 0.01. **E.** *in vivo* limiting dilution tumor formation assay were performed to assess the cancer stem cell potential of AR. Br3CT or AR cells intracranially implanted and sacrificed at same time. H&E staining of mice brain which were implanted Br3CT or AR 1000 cells showed tumor formation and size. Tumorigenic incidences were calculated by rate of tumor bearing mice numbers into total mice numbers per group. Bar represents 2 millimeters. **F.** Expressions levels of EMT and CSC markers of GBM in Br3CT or AR cells were determined by real-time RT-PCR analysis. Data are means ± SE (*n* = 3). **p < 0.01 and ***p < 0.001. **G.** Phenotypes of tumor observed by H&E staining and margins of AR tumor were magnified (right). Arrows indicate tumor cells which were invaded into adjacent normal brain parenchymal. Bar represents 100 microns. **H.** Transwell invasion assays in Br3CT and AR cells were performed. Cells which were passed transwell were counted (right bar graph) after H&E staining (left). Bar represents 200 micron. Data are means ± SE. *p < 0.05.

A subset of GBM patients receiving Bevacizumab initially responded but inevitably succumbed to this disease often with more invasive and aggressive tumor growth pattern. To establish the model systems that can mimic these *Bevacizumab*-mediated therapeutic responses in human patients, we have developed *in vivo* serial transplantation models of *Bevacizumab*-treated GBM. U87MG glioma cell line generates orthotopic xenograft tumors efficiently and expresses a high level of human VEGF. Mice with Bevacizumab therapy were survived significantly longer than the control group (median survival days, 28 days in control group and 48 days in *Bevacizumab*-treated group, *p* < 0.0001) (Figure 1A). Immunohistological analysis using endothelial marker CD34 antibody showed a significantly low vessel density in *Bevacizumab*-treated mice (28.3 ± 8.3% compared to the control, *p* < 0.05) (data not shown). These findings suggest that although Bevacizumab do not target mouse VEGF, tumor-driven human VEGF is a main driver of tumor angiogenesis in this model. In contrast to well-demarcated control tumor, many tumor cells from *Bevacizumab*-treated mice invaded adjacent brain parenchyma (Figure 1B), suggesting an acquisition of more invasive tumor growth pattern by Bevacizumab treatment.

To further characterize these acquired tumor phenotypes by Bevacizumab, we performed the serial transplantation experiments. Tumor cells were isolated from *Bevacizumab*-treated tumor-bearing mice and injected into the new recipient mice orthotopically. These mice were treated with Bevacizumab as well, and the resultant tumors were re-implanted for other recipient mice. We obtained the tumors established by three consecutive *in vivo* passages with Bevacizumab and designated as AR (Avastin-Resistant) tumors. As a control for serial transplantation process, *Bevacizumab*-naïve U87 cells were serially transplanted *in vivo* without Bevacizumab treatment (Br3CT). We first determined the tumor latency by survival analysis of tumor-bearing mice. The median survival of Br3CT orthotopic xenograft mice was 27 days, similar to the parental U87 tumor-bearing mice (Figure 1C). Notably, most of AR tumor-bearing mice died within 20 days with a median survival of 18 days. Next, we determined the Bevacizumab response in Br3CT and AR tumor models. Survival benefits were calculated by the extended survival days by Bevacizumab treatment compared to the untreated control. Mice implanted with Br3CT revealed *Bevacizumab*-mediated survival benefits (2.3 ± 0.06 folds increase), almost identical to the parental U87 tumor-bearing mice. In sharp contrast, AR tumor-bearing mice revealed significantly diminished survival benefit from Bevacizumab treatment (1.6 ± 0.05 folds, *p* < 0.01) (Figure 1D). Collectively, these data suggest that AR tumors grow more aggressively in a *Bevacizumab*-resistant manner.

### *Bevacizumab*-resistant tumors are enriched with tumor initiation capacity and invasiveness

As AR tumors grow more aggressive and faster than the controls, we determined whether these tumors contain higher tumor initiation capacity. *In vivo* limiting dilution tumor formation assay is the most, if not the only, robust functional assay for determining GBM initiation capacity *in vivo*. While Br3CT cells required at least 10000 cells for tumor formation, 1000 cells of AR cells were sufficient to generate orthotopic tumors (Figure 1E). A half of mice injected with only 100 AR cells developed tumors, in contrast to no tumor with Br3CT cells, suggesting that AR tumors are enriched with tumor initiating cells. As the enrichment of stem cell associated markers such as CD133 and SOX2 correlated with tumor initiation capacity, we then determined the expression of these genes. AR tumors express significantly high expression of CD133 and SOX2 than Br3CT control tumors [CD133, 9.2 ± 0.84 fold (*p* < 0.001); SOX2, 6.15 ± 1.8 fold (*p* < 0.01)] (Figure 1F). Next, we determined invasive growth pattern in AR tumor. Histological analysis showed that AR tumors harbored a highly infiltrative and invasive growth pattern *in vivo*, consistent with *Bevacizumab*-treated U87 parental tumors (Figure 1G). To further determine invasive characteristic of AR cells, we isolated tumor cells and processed for *in vitro* matrigel invasion assays. Compared to the BR3CT cells, AR cells harbor more than 3 folds of invasive cells (*p* < 0.05), suggesting that AR tumors are highly enriched with invasive capacity (Figure 1H). As acquisition of mesenchymal properties through EMT-like process is implicated in GBM cell motility and invasiveness, we determined the levels of the representative EMT markers in AR tumors. Expression levels of the representative mesenchymal markers, vimentin and ZEB1, are increased, while expression of the epithelial marker E-cadherin was decreased in AR tumors compared to Br3CT tumor (Figure 1F). Taken together, these data strongly suggest that AR tumors are highly enriched with tumor initiation capacity and invasive growth pattern.

### TLN1 was highly expressed in *Bevacizumab*-treated GBM

**Figure 2:**
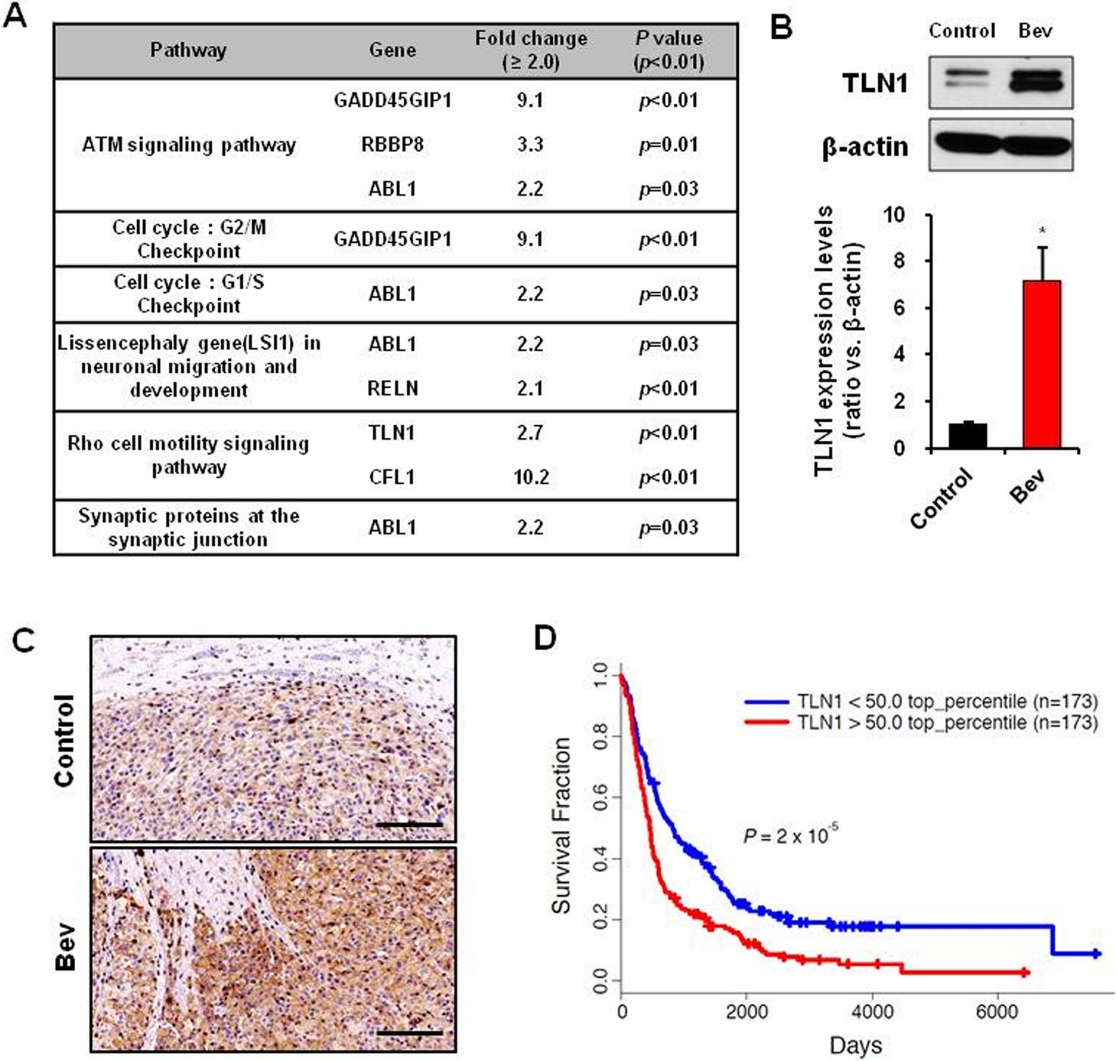
TLN1 expression was increased by bevacizumab treatment in GBM **A.** Pathway analysis of deferent expression genes of mice orthotopically implanted U87MG treated with bevacizumab. **B.** Immunoblots of TLN1 in orthotopic xenograft tumor with or without betacizumab treatment (up). TLN1 expression levels were measured by density of protein bands (down). Data are means ± SE. *p < 0.05. **C.** IHC of TLN1 in orthotopic xenograft tumor with or without betacizumab treatment. Bar represents 200 microns. **D.** Rembrandt Kaplan-Meier survival curves of GBM patients with low or high expression levels of TLN1.

To get molecular insights for *Bevacizumab*-resistance in GBM, we conducted mRNA microarray experiments using U87MG orthotopic xenograft tumors with or without Bevacizumab treatment (*n* = 3 for each group) (Supplementary Table S1 and S2). Pathway analysis using Biocarta database revealed that ATM signaling, cell cycle, neuronal development and Rho cell motility pathways were significantly upregulated in *Bevacizumab*-treated group compared to the untreated group (Figure 2A). As the acquisition of more invasive phenotype is a key characteristic of *Bevacizmab*-resistant GBM, we chose to further investigate the role of the cytoskeleton protein Talin1 (TLN1) that was implicated in cell motility signaling pathways. TLN1 mRNA in *Bevacizumab*-treated group was about 3 fold higher than that in control. Immunoblot analysis showed that levels of TLN1 protein in *Bevacizumab*-treated tumors were > 7 fold higher than those in the control group. (Figure 2B). Immunohistochemical analysis also showed that TLN1 was significantly increased in *Bevacizumab*-treated xenograft tumors (Figure 2C).

In addition, TLN1 was significantly overexpressed in glioma specimens, and its expression correlated with poor survival of glioma patients, determined by Rembrandt databases (Figure 2D).

### Loss of TLN1 diminished clonogenic growth, cell motility, and expression of mesenchymal and stem cell associated markers in GBM cells

**Figure 3:**
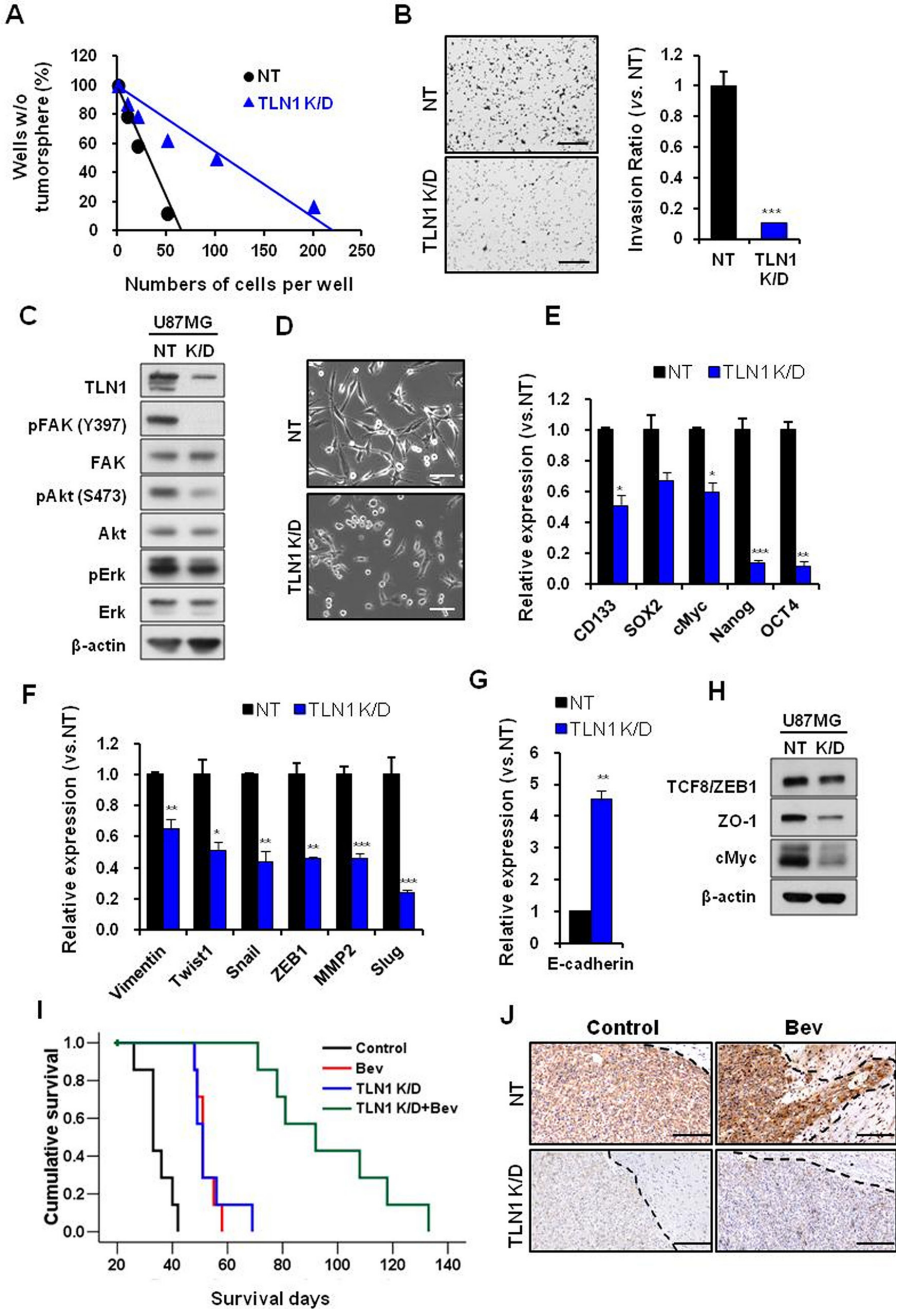
Effects of TLN1 inhibition on malignant progression and survival gains by bevacizumab in U87MG **A.** and **B.** Effects of TLN1 inhibition on clonogenic growth and invasiveness of U87MG. *In vitro* LDA (A) and transwell invasion assay (B) results were shown. Bar represents 200 microns. Data are means ± SE. ***p < 0.001. **C.** Immunoblots of TLN1 and related proteins in TLN1 K/D U87MG. **D.** Cell morphologies were observed by light microscope. Bar represents 20 microns. **E–H.** EMT and CSC markers mRNA expression levels in TLN1 K/D U87MG were determined by real time RT-PCR analysis (E and F). E-cadherin, MET marker, mRNA expression was determined by real-time RT-PCR (G) Immumoblots of EMT and CSC proteins in TLN1 K/D U87MG (H) Data are means ± SE. *p < 0.05, **p < 0.01 and ***p < 0.001. **I.** Kaplan-Meier survival curves of mice (*n* = 10 for each group) orthotopically implanted with U87MG NT or TLN1 K/D U87MG and each groups were divided into two groups by bevacizumab treatment or not. **J.** IHC of TLN1 in tumors of NT or TLN1 K/D U87MG implanted mice with or without bevacizumab treatment. Bar represents 200 microns. Dotted lines indicate margins of tumor.

To determine the functional roles of TLN1 in GBM, we employed shRNA-mediated TLN1 K/D approach. We overexpressed TLN1 shRNA in U87 cells and we determined the role of TLN1 in clonogenic growth by performing *in vitro* limiting dilution assays. Notably, TLN1 K/D cells were inefficient in generating clones compared to the control (Figure 3A). Then, we determined the effect of TLN1 in glioma migration/invasion. Results showed that invasive capacity of U87MG was inhibited approximately 90% by TLN1 K/D (Figure 3B). Collectively, these data showed that TLN1 K/D diminished the clonogenic growth and invasiveness of GBM.

TLN1 was previously implicated in cell migration, mainly through focal adhesion kinase pathway. To determine the alteration of downstream effectors, we performed immunoblots using antibodies against FAK, Akt, and Erk. TLN1 K/D significantly decreased the levels of the phosphorylated of FAK (Y397), and to lesser degree, phosphorylated Akt (S473) and Erk (Figure 3C). In addition, the morphology of TLN1 K/D cells became round and polygonal compared to the parental cells, raising the possibility that TLN1 stimulates mesenchymal properties of GBM cells (Figure 3D).

As TLN1 loss impeded stem-like clonogenic growth and invasive capacities of GBM cells, we then determined the mRNA levels of stem cell associated factors and regulators of invasion and mesenchymal properties. Notably, expression levels of stem cell associated factors including CD133, cMyc, Nanog, and Oct4 were significantly decreased by TLN1 K/D (Figure 3E). While E-cadherin mRNA expression was increased (Figure 3G), the levels of mesenchymal regulators such as vimentin, snail and ZEB1, and MMP2 were significantly decreased in TLN1 K/D cells compared to the control (Figure 3F). These trends were confirmed by the immunoblot analyses (Figure 3H). These data support a key role of TLN1 in regulation of stem-like properties and invasiveness in GBM.

### Loss of TLN1 attenuated resistance to Bevacizumab treatment

Having shown the role of TLN1 in GBM cells *in vitro*, we attempted to address the role of *in vivo* tumor propagation and, more importantly, *in vivo Bevacizumab*-resistance. U87 cells either expressing the non-target (NT) shRNA or *TLN1* shRNA were injected into mouse brains. Without Bevacizumab, mice with TLN1 K/D tumor survived significantly longer than control group (median survival days; Control, 33 days; shTLN1, 51 days, *p* < 0.001). Notably, mice with TLN1 K/D tumor and Bevacizumab survived more than 3 months (median survival days; Bev, 51 days; shTLN1+Bev, 92 days, *p* < 0.001) (Figure 3I). Importantly, histological analysis also revealed that TLN1 K/D tumors do not have protruding invasive cells at the margin despite Bevacizumab treatment, suggesting that TLN1 K/D negates *Bevacizumab*-induce invasive tumor growth pattern (Figure 3J).

### Inhibition of TLN1 attenuated epithelial mesenchymal transition and cancer stem cell properties in AR cells

**Figure 4:**
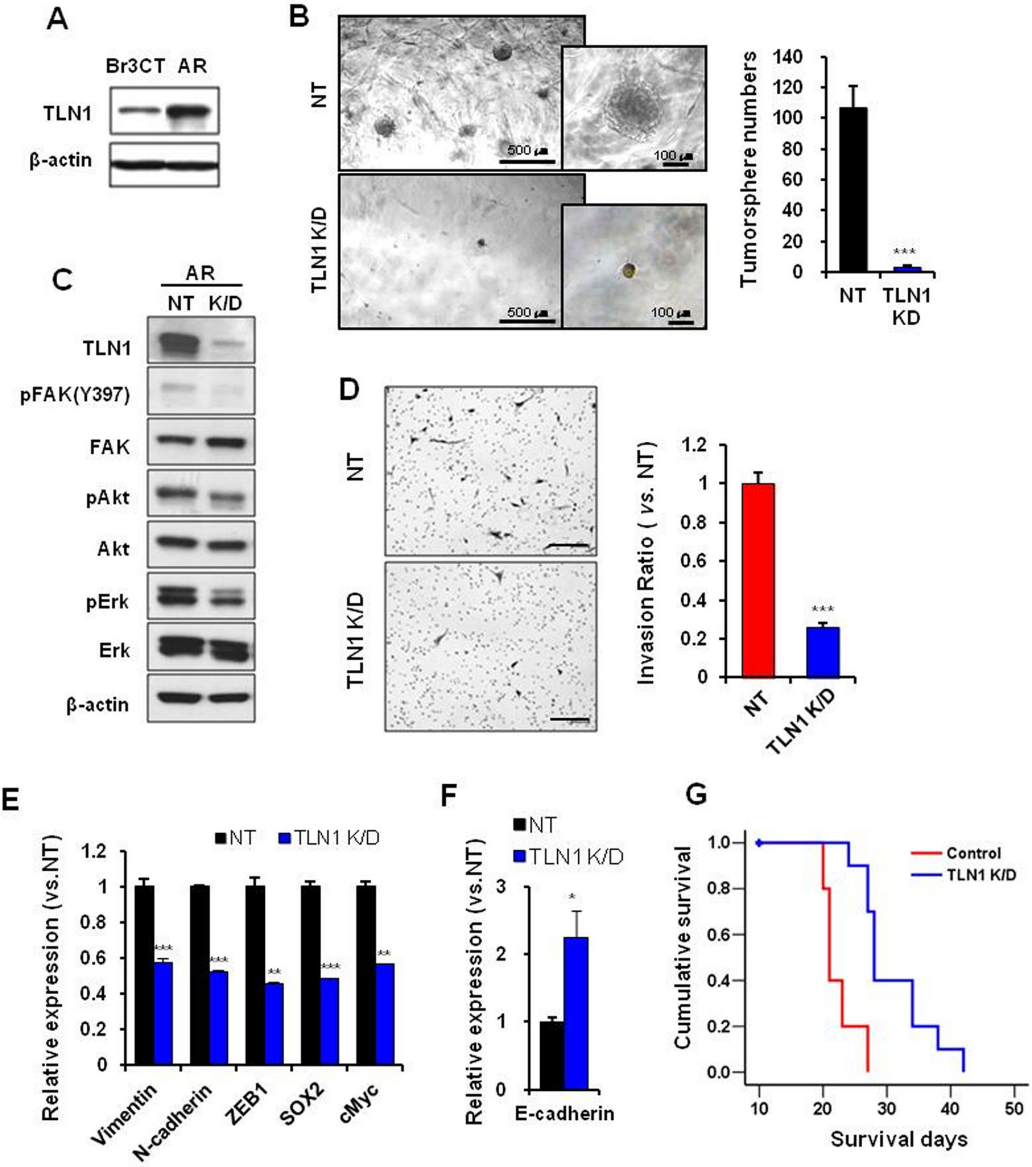
Effects of TLN1 inhibition on acquired malignant progression by bevacizumab treatment **A.** Immunoblots of TLN1 in Br3CT or AR cells. **B.** Tumorsphere forming potentials of TLN1 K/D AR cells were determined by matrigel sphere forming assay. The numbers of tumorsphere were counted. Data are means ± SE. ***p < 0.001. **C.** Immunoblots of TLN1 and related proteins in TLN1 K/D AR. **D.** Transwell invasion assays in NT or TLN1 K/D AR cells were performed. Cells which were passed transwell were counted (right bar graph) after H&E staining (left). Bar represents 200 micron. Data are means ± SE. ***p < 0.001. **E.** Expressions levels of EMT and CSC markers of GBM in NT or TLN1 K/D AR cells were determined by real-time RT-PCR analysis. Data are means ± SE (*n* = 3). **p < 0.01 and ***p < 0.001. **F.** E-cadherin, MET marker, mRNA expression was determined by real-time RT-PCR. Data are means ± SE (*n* = 3). *p < 0.05. **G.** Kaplan-Meier survival curves of mice (*n* = 10 for each group) orthotopically implanted with NT or TLN1 K/D AR. *p* < 0.001.

As the above data suggest the involvement of TLN1 in stem-like characteristics, invasion, and *Bevacizumab*-mediated invasive growth of GBM, we investigated the role of TLN1 in *Bevacizumab*-resistant AR tumors. Consistent with our hypothesis, TLN1 expression was significantly high in AR tumors compared to Br3CT tumors (Figure 4A). Using TLN1 K/D approach, we interrogated the effects of TLN1 in clonogenic growth, stem cell marker genes, and invasion. Similar to the case of TLN1 K/D in parental U87, Inhibition of TLN1 significantly attenuated all of the above (Figure 4B–4F). Furthermore, TLN1 K/D significantly delayed growth of highly aggressive AR tumor (median survival, NT shRNA-AR, 21 days and shTLN1-AR, 28 days, *p* < 0.001) (Figure 4G). Taken together, these results strongly implicate that TLN1 positively stimulate stem-like properties and invasion, and inhibition of TLN1 can diminish *Bevacizumab*-mediated aggressiveness in GBM.

### Functional validation of TLN1 in the patient-derived primary GBM cells with Bevacizumab therapy

**Figure 5:**
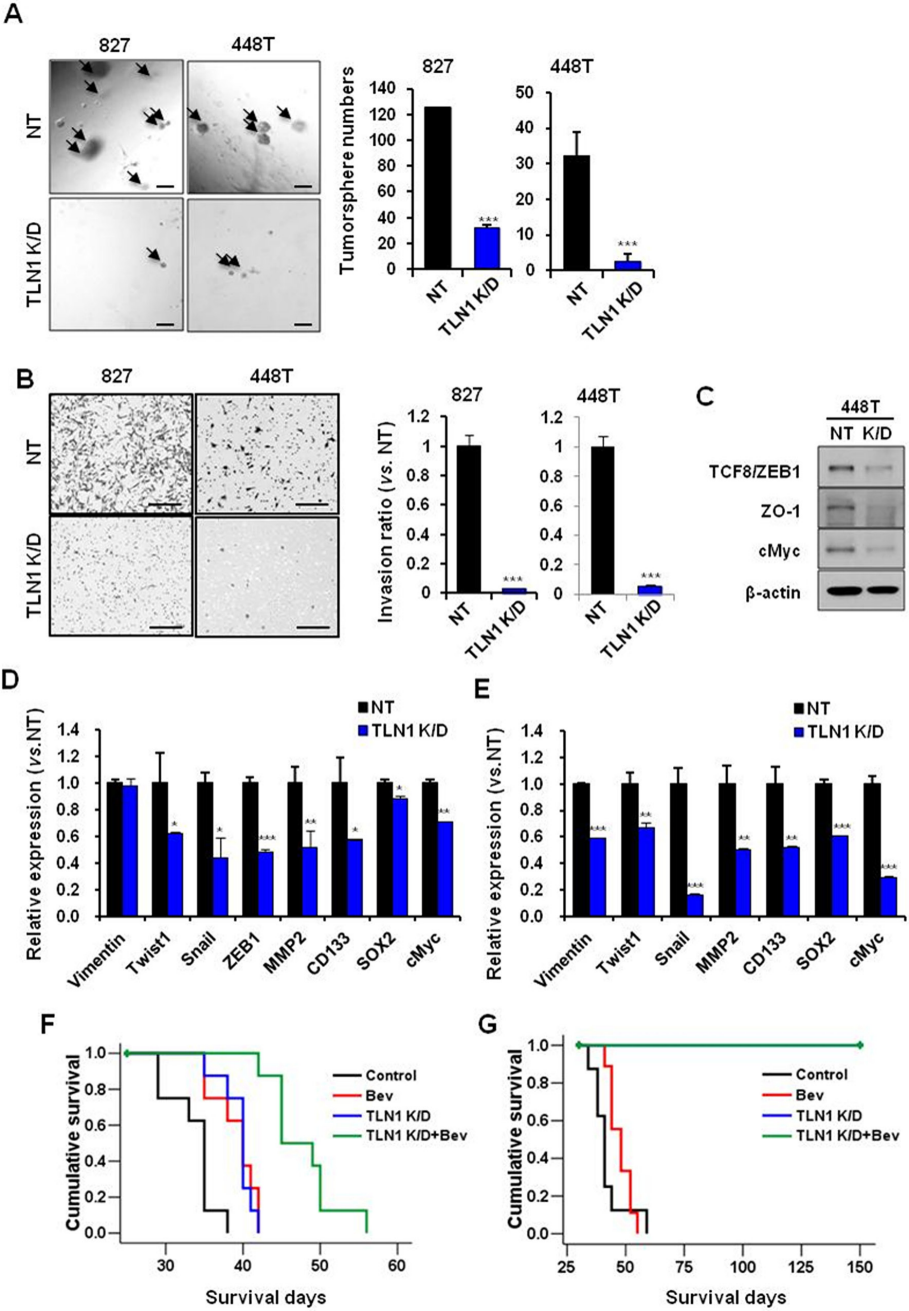
Effects of TLN1 inhibition on malignant progression and survival gains by bevacizumab in patient derived GBM cells **A.** Tumorsphere forming potentials of TLN1 K/D patient GBM cells were determined by matrigel sphere forming assay (Left). Bar represents 100 microns. The numbers of tumorsphere were counted (Right). Data are means ± SE. ***p < 0.001. **B.** Transwell invasion assays in NT or TLN1 K/D patient derived GBM cells were performed. Cells which were passed transwell were counted (Right) after H&E staining (Left). Bar represents 200 micron. Data are means ± SE. ***p < 0.001. **C.** Immumoblots of EMT and CSC proteins in TLN1 K/D patient derived GBM cells. **D.** and **E.** Expressions levels of EMT and CSC markers of GBM in NT or TLN1 K/D in patient derived GBM cells [827 (D) and 448T (E)] were determined by real-time RT-PCR analysis. Data are means ± SE (*n* = 3). *p < 0.05, **p < 0.01 and ***p < 0.001. **F.** and **G.** Kaplan-Meier survival curves of mice (*n* = 10 for each group) orthotopically implanted with NT or TLN1 K/D patient derived GBM [827 (F) and 448T (G)] and each groups were divided into two groups with bevacizumab treatment or not.

Recently, we have shown that xenograft tumors derived from primary GBM specimens recapitulate the patient-specific responses to therapies such as radiation, temozolomide and Bevacizumab treatment [19]. To validate the role of TLN1 in these clinically relevant GBM models, two patient-derived orthotopic tumor models were established and performed *in vivo* Bevacizumab treatment. Similar to AR tumors, TLN1 K/D in both 827 and 448 cells significantly diminished the frequencies of spheres in 3D matrigel limiting dilution assays, raising the possibility that TLN1 function might be associated with extracellular matrix (Figure 5A). Invasion capacities of both cells were significantly decreased by TLN1 K/D (Figure 5B). Immunoblots and RT-PCR analysis showed that TLN1 K/D greatly decreased the expression levels of the stem cell associated factors and EMT markers, further establishing the role of TLN1 in GBM self-renewal and invasion (Figure 5C–5E).

The above data collectively suggest that TLN1 positively activates the process of stem cell renewal and invasion, characteristics that are associated with *Bevacizumab*-resistance. We hypothesized that TLN1 targeting would potentiate *Bevacizumab*-mediated anti-tumor effects in patient-derived GBM tumors. Tumor cells with either NT shRNA or TLN1 shRNA were transplanted for orthotopic tumors, and tumor-bearing mice were treated with Bevacizumab. A striking survival benefit was observed in both models. Inhibition of TLN1 in 827 tumors prolonged the survival, and greater survival gain was achieved by combination treatment [median survival days; Control, 35 days, Bev, 40 days (*p* < 0.01 *vs*. Control), shTLN1, 40 days (*p* < 0.001 *vs*. Control) and shTLN1 plus Bev, 45 days (*p* < 0.001 *vs*. Control) (*p* < 0.001 *vs*. Bev) (*p* < 0.001 *vs*. shTLN1)]. Inhibition of TLN1 in 448T tumors couldn't make tumors for more than 5 months (median survival days; Control, 45 days and Bev, 45 days). These data showed TLN1 targeting strongly inhibited tumor formation and potentiated the effects of anti-angiogenic therapy in patient-derived xenograft models (Figure 5F and 5G).

## DISCUSSION

*Bevacizumab*-mediated anti-angiogenic therapies have met with limited success in the recent large clinical trials. Accumulating evidence suggests that tumor adaptations in VFGF-independent microenvironment, which often includemore aggressive and mesenchymal-liketransitions, are major reasons for transient therapeutic benefit by Bevacizumab [14, 20–24]. Understanding of such resistance mechanisms and identification of molecular targets will lead to the development of more efficacious and sustainable therapeutic strategies. By developing *in vivo Bevacizumab-* resistant tumor models and utilizing a series of patient-derived GBM models, we have identified and validated TLN1 as a key mediator of *Bevacizumab*-resistance in GBM.

The development of the optimal model systems that mimic the responses of the VEGF-neutralizing antibody Bevacizumab remains a critical, unresolved issue. Xenograft models derived from human glioma cell lines and patient-derived primary GBM models can inform the biology of human GBM, however, VEGF neutralization is likely incomplete because Bevacizumab can block only human-derived VEGF but not the murine VEGF. While various mouse glioma models and the use of anti-murine VEGF antibody can circumvent this issue, it is unclear whether murine GBM models can fully recapitulate the biology of human GBM. By a serial transplantation approach, we have developed the tumor models that are enriched with *Bevacizumab*-resistant tumor phenotypes. Consistent with a high level of tumor-derived VEGF in U87, Bevacizumab treatment on the naïve-U87 tumors significantly prolonged the survival of tumor-bearing mice. However, survival benefit by Bevacizumab was significantly decreased by *in vivo* serial transplantations of *Bevacizumab*-treated tumors. Indeed, the development of *Bevacizumab*-resistance over time may represent the therapeutic responses seen in some patients with Bevacizumab. Notably, *Bevacizumab*-resistant AR tumors obtained by three consecutive *in vivo* passages were highly enriched with stem cell features, mesenchymal properties, and invasiveness. Further investigation is required to determine whether enriched stem-like phenotypes in AR tumor is due to clonal selection or enrichment of stem-like populations. It remains incompletely understood how anti-angiogenic therapy affects tumor hierarchy. AR tumors will be therefore an excellent model system to identify molecular mechanisms of evasive resistance and test the efficacies of the combinatory therapeutic approaches that might substantiate anti-angiogenic therapy.

Using these U87-derived *Bevacizumab*-resistant tumor and the patient-derived primary GBM models, we identified that TLN1 was significantly upregulated by Bevacizumab treatment. TLN1 loss inhibited the expression of stem cell-associated proteins and mesenchymal proteins, and impeded invasion and *in vivo* tumor growth. More importantly, Inhibition of TLN1 significantly potentiated the therapeutic effect of Bevacizumab.

Anti-angiogenic effects on cancer stemness and invasiveness are possibly related to the hypoxia generated in the tumor microenvironment [25]. The transcription factor prediction databases such as Match Program (www.gene-regulation.com) and JASPAR database (http://jaspar.genereg.net) revealed that hypoxia inducible factor 1β (HIF1β) is a potential transcriptional factor of TLN1 (data not shown). Therefore, in the hypoxic condition, HIF1β might stimulate TLN1 mediating-intracellular signaling pathways combining with HIF1α. Because anti-angiogenic therapies induce not only hypoxia but also metabolic stress, low nutrient condition could be also one of the possible regulating mechanisms of TLN1 in the GBM.

TLN1 is known as a molecule involved in maintaining cytoskeleton and integrin signaling [26]. Recent studies have demonstrated that the interaction between TLN1 and integrin is an early event in membrane–cytoskeleton cross-linking and that Inhibition of TLN1 could prevent integrin activation, suggesting a role of TLN1 as an upstream regulator of integrin pathway [27–30]. As TLN1 was reported to regulate the cell–cell adhesion protein E-cadherin in an integrin-independent manner, TLN1 appears to regulate multiple downstream effectors [30]. Recently, Integrin β1 was implicated to mediate *Bevacizumab*-resistance in GBM [26]. It is possible that TLN1 and integrin β1 in GBM are involved in the same signaling axis. In line with this, reciprocal communication between extracellular matrix and tumor cells is recognized as a major tumor microenvironment and TLN1 are known to be a key regulator in this process. Alternatively, TLN1 in GBM may contribute to invasion of cancer cells via survival signaling pathways by activating ECM-integrin–mediated signaling and promoting anoikis resistance [31]. Further study needs to demonstrate theregulating mechanismsof TLN1 regarding *Bevacizumab*-resistance.

Although exact pathway to which TLN1 is driving stem-like invasive tumor phenotypes and *Bevacizumab*-resistance is yet to be determined, potent effects of TLN1 targeting on GBM cell growth and *Bevacizumab*-resistance strongly supports TLN1 as a potential therapeutic target.

In conclusion, we demonstrate that TLN1 is a critical regulator of stem-like features, invasion, and *Bevacizumab*-resistance in GBM. Furthermore, TLN1 is a potential anti-GBM target alone or in combination with anti-angiogenic therapy.

## MATERIALS AND METHODS

### GBM patient-derived primary cell culture and cell line

Following informed consent, surgical specimens were obtained from the GBM patient who had brain tumor removal surgery at the Samsung Medical Center (Seoul, Korea) in accordance with the appropriate Institutional Review Boards. Genomic and molecular alterations of these tumors were previously reported. Dissociated GBM cells were cultured in neurobasal media with 0.5X, N2 supplement (17502-048, Invitrogen) and 0.5X, B27 supplement (12587-010, Invitrogen), 25 ng/ml, human recombinant basic fibroblast growth factor (bFGF) (233-FB-01M, R&D system) and 25 ng/ml, epidermal growth factor (EGF) (236-EG-01M, R&D system). U87MG cell line was obtained from and authenticated by American Type Culture Collection. Cells were maintained in Minimum Essential Media (MEM) (11095, Gibco) supplemented with 10% fetal bovine serum (FBS) (12483-030, Gibco) and antibiotics, penicillin/streptomycin (15140-122, Gibco).

### GBM orthotopic xenograft models

The animal experiments were approved by the Review Board of Samsung Biomedical Research Institute (Seoul, Korea). For establishment of human GBM orthotopic xenograft, six-week old female BALB/c nude mice (Orient Bio) were used. GBM cells in 5 μl Hank's Balanced Salt Solution (GIBCO) were directly implanted into the brains of anesthetized mice using rodent stereotactic frame [co-ordinates: anterior/posterior +1.0 mm, medial/lateral +1.7 mm, dorsal/ventral −3.2 mm]. Bevacizumab (10 mg/kg, twice a week, intraperitoneal injection) treatment was started at 1 week after tumor cell implantation. The reduction of the total body weight (>20%) was regarded as mortality.

### Gene expression profiling

Microarray gene profiling was conducted using Agilent Sure Print G3 Human GE 8x60k Microarray, according to the manufacturer's instruction. Raw data were generated from scanned images using Agilent Feature Extraction Software. FE (feature extraction) files were processed and normalized, using Agilent Genomic Work Bench with 028004_D_F_20100430.xml design file.

### Short hairpin RNA-mediated TLN1 knockdown

MISSION ® shRNA pLKO.1-puro plasmid DNA vectors targeting TLN1 (NM_006289, Sigma-aldrich) and shRNA non-target control (SHC002V, Sigma-aldrich) were purchased from Sigma. (USA). Lentiviral shRNA was produced by co-transfection of the lentiviral packaging mix, including VSV-G, pRSV-Rev, and pMDKg/pRRE, with a shRNA transfer vector into HEK 293T packaging cells and the viral supernatant was collected to transfect U87MG, U87-AR, 827 and 448T followed by puromycin selection. The cells stably expressing shRNA were maintained in puromycin.

### Quantitative real-time RT-PCR

Total RNA extraction was performed using RNeasy Plus Mini kit (74134, Quiazen) and RNA concentration was measured by Nano Drop 2000 (Thermo Fisher Scientific). cDNA was sunsequently synthesized using SuperScript^TM^ III First Strand Synthesis System (18080–051, Invitrogen) for quantitative real-time RT-PCR. Diluted cDNA and gene-specific primers (Table 1) were mixed with 5X HOT FIREPol® EvaGreen ® qPCR Mix Plus (ROX) (Solis Biodyne) according to the supplied protocol. Amplification was performed in the following temperature-time profile: 95°C −10 minutes, then 40 cycles; 95°C −15 seconds and 60°C −1 minute. The levels of expression for the selected genes were normalized by expression of beta-actin.

**Table 1:** Primer sequences

TLN1	F: CCCTGATGTGCGGCTTCG R:TGTCCTGTCAACTGCTGCTTC
ZEB-1	F: TTCAAACCCATAGTGGTTGCT R: TGGGAGATACCAAACCAACTG
N-cadherin	F: ACAGTGGCCACCTACAAAGG R: CCGAGATGGGGTTGATAATG
Vimentin	F: GTTTCCAAGCCTGACCTCAC R: GCTTCAACGGCAAAGTTCTC
Twist1	F: CGGACAAGCTGAGCAAGATT R: CCTTCTCTGGAAACAATGAC
Snail	F: CTTCCAGCAGCCCTACGAC R:CGGTGGGGTTGAGGATCT
E-cadherin	F: CAGCACGTACACAGCCCTAA R: ACCTGAGGCTTTGGATTCCT
CD133	F: TCCACAGAAATTTACCTACATTGG R: CAGCAGAGAGCAGATGACCA
SOX2	F: TGCTGCCTCTTTAAGACTAGGAC R: CCTGGGGCTCAAACTTCTCT
cMyc	F: AAAACCAGCAGCCTCCCGCG R: GGGTGGGCAGCAGCTCGAAT
B-actin	F: AAAATGGCAGTGCGTTTAG R: TTTGAAGGCAGTCTGTCGTA

### Western blotting

Western blots were conducted as described previously [32], using primary antibody to TLN1 (ab71333, Abcam), p-FAK (Y397) (8556, Cell signaling), FAK (3285, Cell signaling), p-Akt (S478) (9271, Cell singling), Akt (9272, Cell signaling), MAPK (9102, Cell signaling), p-MAPK (9101s, Cell signaling), Sox2 (3579, Cell signaling), Vimentin (5741, Cell signaling), N-cadherin (4061, Cell signaling), ZO-1 (8193, Cell signaling), Snail (3879, Cell signaling) and TCF8/ZEB1 (3396, Cell signaling).

### *In vivo* limiting tumor formation assay

For validation of *in vivo* tumor forming ability of GBM cells, orthotopic xenograft models were established by injection of different numbers of tumor cells like as 100, 1000 and 10000 cells orthotopically. All mice were sacrificed, simultaneously. For evaluate morphology of tumor, the brain slices were fixed in 10% formalin/PBS, embedded in paraffin, sectioned into 4 μm coronal sections, and stained with H&E.

### *In vitro* limiting dilution assay

Limiting dilution assay was performed in 96 well plates. Briefly, GBM cells were seeded (1 to 500 cells/well) in 8 wells at each cell numbers. After 2 or 3 weeks, wells without spheres were counted and analyzed.

### Three-dimensional (3D) sphere forming assay in matrigel

The TLN1 knockdown (K/D) GBM cells (5 × 10^2^ cells of each cells) were mixed with matrigel (BD Bioscience) and poured into the 4 well plates. After 2 or 3 weeks, spheroid were counted and compared with NT of each cells.

### Two-dimensional (2D) invasion assay

Matrigel invasion assays were performed at 37°C for 24 or 48 hours using transwell membrane coated with matrigel (354483, BD Bioscience). NT or TLN1 K/D of U87MG cell, AR cell and patient derived GBM cells, 827 and 448T, were seeded onto the upper wells of precoated transwells, 1 × 10^4^ cells per well. Lower wells of the transwells contained the same medium with 1% FBS. After 24 or 48 hours of incubation, the cells on the upper well and the membranes coated with Matrigel were swabbed with a Q-tip, fixed with methanol, and stained with hematoxylin and eosin (H&E). The cells that penetrated through filter were counted at a magnification of ×200 in 10 randomly selected fields, and the mean number of cells per field was recorded.

### Histological analysis

Immunohistochemical analysis was performed as described [33] and conducted using antibodies against TLN1 (ab71333, Abcam) and CD34 (ab8158, Abcam).

### Statistical analysis

Statistical comparisons were performed using the Student's *t*-test. Survival analysis was performed using the Kaplan-Meier and the log-rank tests. Multivariate analyses were done with ANOVA with LSD tests. *p*-values < 0.05 were considered to indicate a statistically significant result.

## SUPPLEMENTARY TABLES






