# Supplementary material for: Talin1 targeting potentiates anti-angiogenic therapy by attenuating invasion and stem-like features of glioblastoma multiforme
Source: Oncotarget. 2015 Aug 20;6(29):27239–51. doi: 10.18632/oncotarget.4835 (PMC4694986; doi:10.18632/oncotarget.4835)
Supplement: Supplementary file 1 [file oncotarget-06-27239-s001.pdf]

SUPPLEMENTARY TABLES

Supplementary Table S1: Genes up-regulated in the Bevacizumab-treated U-87MG tumors

Supplementary Table S2: Genes down-regulated in the Bevacizumab-treated U-87MG tumors
